# Comments in “Coronary Artery Bypass Grafting Following ST Elevation Myocardial Infarction Outcomes Are Associated With Time to Surgery”

**DOI:** 10.1093/icvts/ivag109

**Published:** 2026-04-09

**Authors:** Bedirhan Buğra Bayıcı, Fatih Kızılyel

**Affiliations:** Department of Cardiovascular Surgery, Koşuyolu High Specialization Training and Research Hospital, Istanbul, 34846, Türkiye; Department of Cardiovascular Surgery, Koşuyolu High Specialization Training and Research Hospital, Istanbul, 34846, Türkiye

To the Editor,

Crane et al^1^ recently reported a large national analysis evaluating outcomes of isolated coronary artery bypass grafting (CABG) performed during the same admission for ST-segment elevation myocardial infarction (STEMI), stratified by time from presentation to surgery. Their observation that in-hospital mortality was highest when CABG was performed within 48 hours (6%) and lowest when surgery occurred between 2 and 15 days (3%) raises an important clinical question regarding the optimal timing of surgical revascularization after STEMI.

However, we believe that interpreting these outcomes calls for careful consideration of potential confounding by indication. In the reported cohort, markers of haemodynamic instability differed substantially between groups. In particular, intra-aortic balloon pump use was markedly higher in the <48-hour cohort compared with later surgical groups.^1^ Such differences strongly suggest that patients undergoing very early surgery may represent a more critically ill population requiring urgent revascularization rather than reflecting an inherent risk associated with surgical timing itself. Similar observations have been reported in prior studies examining CABG timing after acute myocardial infarction.^2^^,^^3^

Administrative datasets also present important limitations when evaluating surgical timing. Crucial clinical variables influencing operative decision-making—such as STS risk score, SYNTAX score, left ventricular ejection fraction, infarct territory, and cardiogenic shock severity—are not captured in these databases. Residual confounding related to these factors may therefore remain despite multivariable adjustment.^4^ In addition, the markedly higher stroke incidence observed in patients undergoing CABG ≥16 days after STEMI may reflect prolonged critical illness, extended mechanical circulatory support exposure, or complex perioperative trajectories rather than the delay itself.

We agree with the authors that their findings show a potentially safer operative window when surgery can be deferred. Nevertheless, the relationship between surgical timing and outcomes after STEMI likely reflects the interaction between patient instability, revascularization strategy, and the evolving clinical course. Additional investigations incorporating granular clinical data and advanced causal inference approaches may better clarify whether timing influences mortality independently or primarily reflects patient selection.^5^^,^^6^

## Data Availability

Not applicable.
